# The immunomodulatory role of human cytomegalovirus in cancer in the era of checkpoint immunotherapy

**DOI:** 10.1098/rstb.2024.0413

**Published:** 2025-11-06

**Authors:** Julia Bremke, Cecilia Jay, Martin Little, Benjamin P. Fairfax

**Affiliations:** ^1^ Department of Oncology, University of Oxford, Oxford OX3 7DQ, UK; ^2^ Universität Leipzig Medizinische Fakultät, 04103 Leipzig, Germany; ^3^ Somerville College, University of Oxford, Oxford OX2 6HD, UK

**Keywords:** cancer, immunotherapy, infection

## Abstract

This review discusses the role of chronic human cytomegalovirus (HCMV) infection in patients receiving immune checkpoint blockade (ICB) treatment against different malignancies, with an especial focus on malignant melanoma. We briefly outline the impact of HCMV infection on the immune system as an entity before exploring its role in the context of cancer. We then address the potential impact on the efficacy of immunotherapeutic agents, particularly ICB, before looking at development of ICB-related autoimmune toxicity. Finally, we discuss potential avenues for future research.

This article is part of the discussion meeting issue ‘The indirect effects of cytomegalovirus infection: mechanisms and consequences’.

## 1. Introduction

Overall immune fitness has a significant impact on the development and progression of cancer [1]. This is evidenced by the higher rates of certain malignancies in immunocompromised individuals and the sensitivity of a subset of tumours to systemic treatment with immune checkpoint blockade (ICB) therapy [2]. Chronic infections induce substantial changes in both innate and adaptive immunity that can impact both an individual’s susceptibility to cancer and their response to treatment. Infection with human cytomegalovirus (HCMV) is particularly associated with profound changes in T cell and natural killer (NK) cell subset composition that may impact immunosurveillance of pre-malignant cells and thus disease trajectory [3,4]. In keeping with a role for HCMV in cancer immunity, HCMV-specific T cells have been identified both peri- and intratumourally [5,6]. Moreover, recent work demonstrates ICB can activate HCMV-reactive cells and demonstrates HCMV plays a protective role against metastatic melanoma (MM) [7]. Given these new findings, the role of HCMV in cancer development and progression, plus interaction with efficacy and toxicity of immunomodulatory anti-cancer treatments, require review and re-interpretation.

## 2. Human cytomegalovirus and immunity

### (a) Human cytomegalovirus leads to disseminated infection

HCMV is a herpes virus (HHV-5), which, in common with other Herpesviridae, causes chronic infection characterized by a phase of latency after primary infection, whereupon the virus persists at virtually undetectable levels for the duration of the host’s lifespan. The competent immune system employs a variety of mechanisms to detect and delete infected cells whilst suppressing HCMV reactivation and dissemination. Although this persistent immune response restricts HCMV to a dormant state within a reservoir of infected cells, HCMV is observed to periodically reactivate, entering a lytic cycle characterized by viral replication and detectable viraemia. The triggers for reactivation are poorly understood but include immune-stressors such as intercurrent infections, as observed in people living with human immunodeficiency virus (HIV) [8], and also seen in immunocompetent individuals. In contrast to other herpesviruses, which have tissue-specific tropisms, for example herpese simplex virus (HSV)-1 and HSV-2, which segregate to neural tissue [9], HCMV infects both parenchymal and connective tissue, including epithelial and endothelial cells, fibroblasts and smooth muscle cells [10,11]. This cellular promiscuity results in HCMV detection across diverse tissues, including salivary glands, the gastrointestinal tract, uterine cervix [12], vascular and lymphatic endothelium [13,14] and peripheral blood mononuclear cells (PBMCs) [15]. Within the blood compartment, CD34${,}^{+}$ haematopoietic stem cells (HSCs) and monocyte progenitors support latent HCMV, giving rise to infected monocytes and macrophages [10]. HCMV does not genomically integrate but instead persists in dividing cells through several molecular mechanisms that maintain the HCMV genome in a latent episome. These include microRNA-mediated signalling as well as subclinical reactivation to ‘reseed’ the HSC reservoir [16]. In sum, HCMV infidelity translates into a disseminated pattern of infection, resulting in a generalized, low-level immune activation that is implicated in modulating inflammatory, autoimmune and neoplastic conditions [17,18].

Globally, immunoglobulin G (IgG) seropositivity to HCMV is approximately 50% across much of Western Europe [19], although seroprevalence in Sweden and Portugual is noted to be at *ca* 80% [20,21]. In Asian countries, seropositivity is typically over 80% [22,23], and in tropical regions and Latin America it is *ca* 95% or above [24,25]. At younger ages, females are more likely to be seropositive than males [19,23,24,26], with parity being independently associated with seropositivity [23]. Interestingly, comparative historical analyses have demonstrated reduced HCMV seroprevalence in countries such as Germany over the last few decades [27]. The causes of this are unclear but may relate to smaller family sizes, reduced over-crowding, lower breast-feeding rates and generalized changes in hygiene practices. Indeed, such a cohort effect is promulgated to underpin the divergence in seroprevalence between the under 40s and later ages in the UK [28]. However, the transmission of HCMV in Western countries is surprisingly poorly described. For example, it is unclear why HCMV does not show similar patterns of seroprevalence to Epstein–Barr virus (EBV—HHV4), which infects *ca* 95% of the UK population; instead, a steady increase in HCMV seroprevalence is observed with age, estimated at *ca* 0.7% per year in the UK Biobank [29]. This might relate to increased susceptibility to primary infection with age, despite potentially reduced exposure. Alternative explanations include a mismatch between detected seropositivity and underlying infection, with some individuals having a prolonged subclinical prodrome. The advent of large cohort studies should hopefully clarify such points, but this is a vital factor to consider when exploring associations between HCMV and cancer, which similarly demonstrates age-related increases in prevalence. Likewise, the strong association between HCMV seropositivity and poorer sociodemographic status, a risk factor for many cancers and a predictor of worse cancer-treatment outcomes, is another serious confounding factor that must be accounted for when exploring associations between HCMV and cancer.

### (b) Human cytomegalovirus has both immuno-stimulatory and suppressive properties

HCMV is a complex virus with a large genome that has co-evolved with humans [30]. This relationship has driven competing viral and host adaptations: HCMV evolving to evade immune surveillance while host immunity has adapted to suppress infection in a latent phase [31,32]. The immune evasion required for persistence of chronic viral infection is analogous to that required by neoplastic cells, which similarly evolve to evade immunity in the establishment of cancer. This co-evolution is such that, in health, the immune system typically remains dominant to HCMV, with circulating virus only detectable in the context of severe immunosuppression. Such a tolerant relationship does not hold in the development of cancer, however, which requires escalatory immune-evasion prior to disease dissemination. Infection of cells with HCMV results in local production of inflammatory mediators and cytokines, including cyclo-oxygenase 2 (COX-2), interleukin (IL)-6, IL-7 and IL-11 [33]. Once infection is established, this inflammatory response may be beneficial for HCMV, with early work detailing tumour necrosis factor (TNF)-mediated NF-*κ*B translocation further activating the HCMV immediate–early (IE) gene promoter, reflecting reciprocal positive feedback [34]. Thus inflammation at sites of HCMV infection, which leads to antigen-presenting cell recruitment, may result in further myeloid cell infection [35]. In keeping with this pro-inflammatory quality, HCMV has been associated with inflammatory disease processes, most notably atherosclerosis and ischaemic heart disease [36], reflecting endothelial tropism of HCMV. HCMV has similarly been implicated in the promotion of autoimmunity via the induction of autoantibodies such as those to phospholipids and CD13 [37]. This may result from viral mimicry and cross-reactivity, as well as through the interaction of HCMV with toll-like receptor 7 (TLR7) and TLR9 in plasmacytoid dendritic cells (pDCs) [38]. The relationship between HCMV infection and poorer sociodemography means that associations with inferior health outcomes are potentially confounded, however [39,40]. Nonetheless, in patients with solid organ transplants (SOTs), vascular sclerosis is clearly associated with HCMV infection, with receipt of organs in HCMV seronegative recipients from HCMV positive donors having a hazard ratio (HR) of 3.1 for thromboembolic events in the subsequent 5 years post-transplant [41]. These observations likely reflect the importance of immune control of endothelial HCMV, which is impaired in immunosuppressed individuals [42,43].

In contrast to these inflammatory properties, HCMV has also evolved diverse mechanisms to suppress immunity and promote host persistence that might be anticipated to interfere with anti-cancer immunity in a pro-tumourigenic fashion [35]. Specifically, the HCMV genome encodes factors that inhibit antigen processing and presentation, mimic or antagonize endogenous cytokines, and prevent apoptosis of infected cells. Key examples include the proteins US2 and US6, which inhibit tapasin (TAP) and TAP-mediated peptide transport [44,45], and US3, which promotes class I major histocompatibilty complex (MHC) endoplasmic reticulum retention [46,47]—all focused on impairing antigen presentation. This forms a corollary with the frequent mutation or loss of heterozygosity of class I MHC proteins observed in the evolution of immuno-evasive tumours [48]. Likewise, HCMV peptides can directly act as homologues and decoy receptors for cytokines, impeding local effector cytokine activity [49]—a key mimic being vIL-10, an IL-10-like protein that similarly acts as a potent anti-inflammatory agent via IL-10R ligation [50]. Notably, vIL-10 can inhibit T helper type 1 (Th1) cytokines, including interferon-gamma (IFN*γ)* and IL-2, and cytokine production by monocytes and macrophages, inhibiting dendritic cell maturation and migration, reducing MHC class II expression and antigen presentation [51]. Conversely, whereas vIL-10 might be anticipated to promote an immune suppressive and tumorigenic microenvironment in HCMV-latent tissues, recent evidence indicates IL-10 is involved in effective anti-tumour immunity, being key to CD8${,}^{+}$ T-cell IFNγ-mediated anti-cancer responses [52] and capable of reversing T-cell exhaustion [53]. Furthermore, several HCMV encoded peptides have been shown to induce IL-10 secreting CD4${,}^{+}$ T cells [54,55]. Thus, this HCMV-modulated cytokine demonstrates context-specific activity with tolerogenic properties in the context of autoimmunity, whilst being directly implicated in anti-tumour responses; whether vIL-10 mimics all diverse actions of IL-10 is unclear. HCMV also encodes pUL21.5, a small peptide that antagonizes the activity of the cytokine CCL5 (RANTES), which, while important in the T-cell response to chronic infection [49,56], has been implicated as a pro-inflammatory mediator of tumour progression [57]. Finally, the ability of HCMV to inhibit apoptosis in infected cells via the product of the gene *UL36*, which prevents caspase-8-mediated apoptosis [58], is of notable relevance, caspase-8 mutations being a frequent finding across cancers, including colorectal [59], gastric [60] and head and neck [61] cancers, where they may be prognostic [62].

### (c) Human cytomegalovirus alters the adaptive landscape

As per other viral infections, HCMV infection elicits the development of virus-specific CD4${,}^{+}$ and CD8${,}^{+}$ T cells [63]. However, the T-cell response to HCMV is anomalous to that towards other viruses, including those that elicit chronic infection [64]. The most prominent difference is hyper-expansion of HCMV-reactive T-cell clones, accompanied by T-cell receptor (TCR) repertoire skewing [65–67]. Over the course of its latency HCMV-triggered T-cell clonal outgrowth is such that 10–20%, and in chronically infected elderly individuals up to 50%, of the total CD8${,}^{+}$ T-cell pool may be HCMV- reactive [68]. This phenomenon is referred to as ‘memory inflation’ and leads to a less diverse and more uneven TCR repertoire. The precise determinants of inter-individual variation in memory inflation are unknown—although murine models, where the inoculating dose of MCMV can be modulated, indicate that the infective titre of HCMV influences ensuing T-cell subset changes [69]. In chronic HCMV infection, virus-reactive T-cell clones develop a stereotypical immune phenotype. Instead of the conventional post-infection transition from effector to lymph node resident central memory T cells (T_CM_), or the formation of an ‘exhausted’ phenotype seen in other chronic viral infections [70], HCMV is characterized by persistence of effector memory (T_EM_) and terminally differentiated effector memory CD45 RA-expressing (T_EMRA_) T-cell expansion, even in the absence of viraemia [3,71,72]. These HCMV-reactive memory T cells exhibit low expression of lymph node homing markers (CD62L, CCR7), consistent with a blood and peripheral tissue distribution. HCMV-reactive T cells also show reduced expression of CD27 and CD28, and higher levels of inhibitory receptors KLRG-1 and LILRB1 (CD85j) but not PD-1 [64]. Crucially, efficient production of IFNγ and TNF is retained [65]. This maintenance of cytotoxic potency forms a central facet of anti-HCMV T-cell phenotype [64]. The other major change in T-cell subsets attributable to HCMV is the expansion of non-Human Leukocyte Antigen (HLA)-reactive Vδ2 negative γδ T cells. In common with αβ T cells, these show oligoclonality in HCMV infection and retention of cytotoxicity [73]. Whereas NK cells are not classically defined as adaptive, the differentiation of specific NK cells in HCMV, referred to as adaptive NK cells, is well described [74]. These cells similarly have a distinct phenotype, with upregulation of CD94/NKG2C and downregulation of markers, including CD161 [74,75]. Importantly, NK cells can control HCMV in the absence of T cells [76]. Thus, whilst NK cells are not the central focus of this review, given their role in tumour immunosurveillance, future consideration of the relationship between HCMV and NK-related anti-cancer effects will be vital.

#### (i) Functional consequences of human cytomegalovirus infection

**Figure 1.**
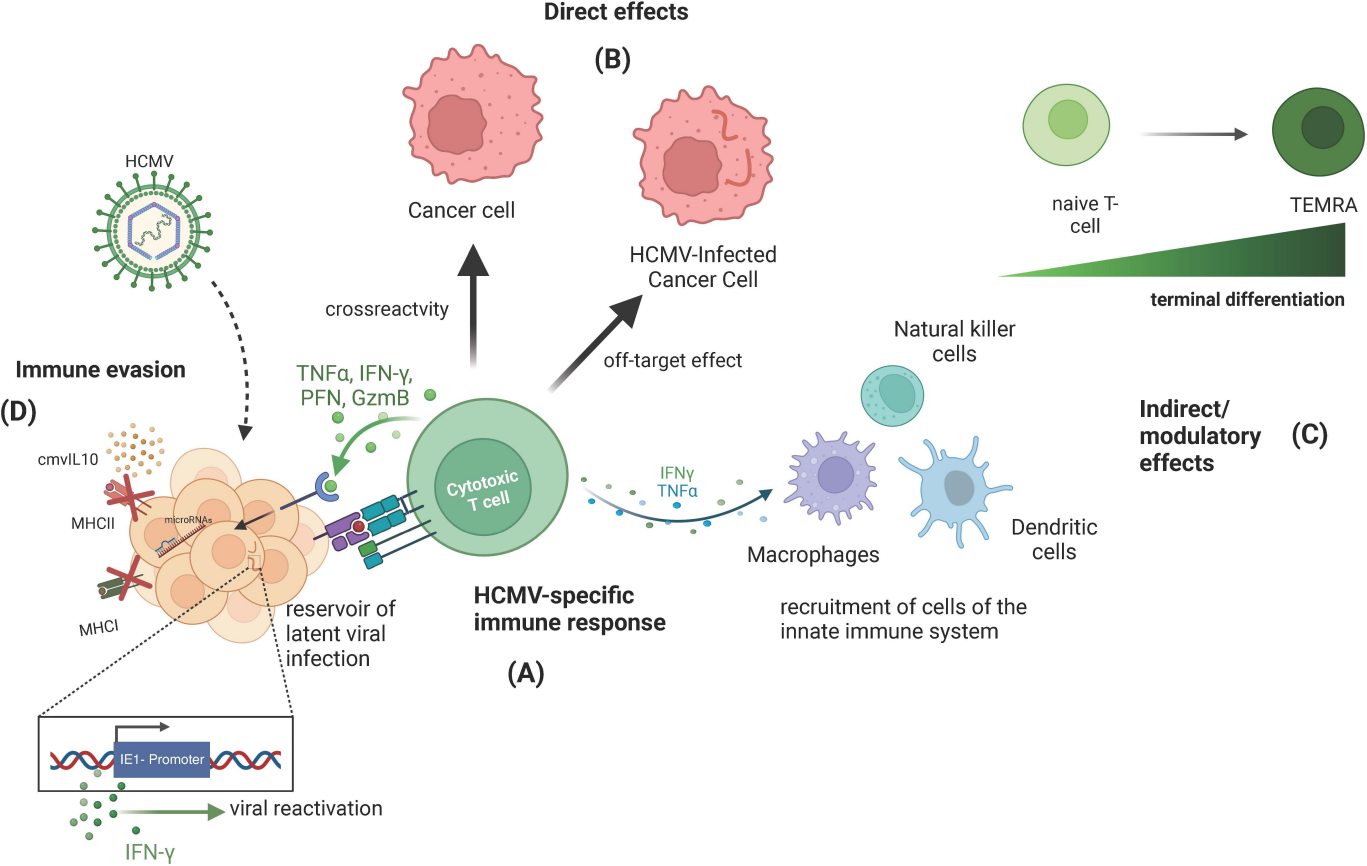
Complexity of direct and indirect effects of human cytomegalovirus (HCMV) and tumour immunity. (A) HCMV infection and subsequent reactivation events elicit a strong cytotoxic T-cell response coupled with proinflammatory cytokine signalling to other innate and adaptive immune-cell subsets. (B) This may directly influence anti-cancer immunity, either through direct targeting of HCMV antigen presentation in HCMV-infected cancer cells, or through cross-reactive epitopes between HCMV and tumour cells. (C) HCMV-reactive T cells may also be involved in bystander effects whereby immunotherapy leads to cytokine release that may have directly anti-tumour effects. (D) HCMV has pleiotropic effects on the immune system and, in some scenarios, may act as an immunosuppressive agent in the tumour microenvironment. TEMRA, effector memory RA. Figure created in BioRender. Please see expansion of abbreviations here: TNFa - 'tumour necrosis factor alpha', IGNg - 'interferon gamma', PFN - 'perforin', GZMB - 'granzyme B', MHCII- 'major histocompatibility complex class II', MHCI - 'major histocompatibility complex class I', cmvIL10 - 'cytomegalovirus interleukin 10'.

Immunological flexibility is an important parameter for health and crucial during de novo infection and the prevention of *in situ* malignancies. HCMV undermines T-cell naivety, with potential consequences when encountering novel antigens. The loss of TCR diversity in HCMV infection, coupled to inflammatory pathway induction, parallels age-related immunosenescence—with a drop-off in thymic output, cellular senescence, mitochondrial dysfunction, and impaired proliferation and antibody production [77]. The immunosenescent phenotype of HCMV has been proposed to compromise cancer immunosurveillance and enhance vulnerability to some neoplasias [78]. However, this theoretical synergistic relationship between HCMV and susceptibility to malignancy has limited epidemiological support (discussed below). In the context of tumour immunology, few studies have specifically addressed the contribution of HCMV. HCMV’s contribution to functional immunosenescence is complex and often contradictory, however, which may reflect divergent properties of HCMV across the lifespan. Whereas chronic HCMV-mediated immune activation likely potentiates immunosenescence in the elderly, evidence exists that HCMV bolsters immune responses to infection or vaccination in younger individuals [32]. Specifically, HCMV has protective, augmentative immune properties within healthy animal responses to infection. Mice infected with MCMV exhibited enhanced IFNγ-dependent responses to subsequent influenza infection compared with MCMV-free mice in a cross-protective manner [79]. The same study assessed immune responses in 91 healthy individuals from two age groups (those 20−30 and 60+) following influenza vaccination, assessing antibody titre, serum cytokines and chemokines, immune cell phenotype and gene expression, as well as cell function. This showed HCMV may additionally amplify the response to vaccination, not only infection. This cross-protection mainly manifested in the younger age group, however [79]. It is thus foreseeable that HCMV-mediated IFNγ augmentation might transfer to other pathogens and cancer undergoing treatment with ICB. In keeping with this, HCMV’s immune-stimulating properties are under exploration as an adjuvant for cancer vaccines or oncolytic therapies, promoting more focused and potent immune responses, particularly in patients with a larger number of HCMV-reactive T cells (figure 1) [80,81].

## 3. Human cytomegalovirus infection in patients with cancer

Whilst HCMV puts an immunological burden on infected individuals, with consequences including reduced T-cell naivety, the evidence that HCMV increases risk of cancer is limited. This partly reflects difficulties in deconvoluting epidemiological confounders, most notably associations with poorer sociodemography, encompassing factors such as over-crowding and smoking rates, from causal effects. It is also the case that cancer-subtype-specific analyses across large populations are limited. One of the stronger epidemiological analyses of HCMV-related mortality is the European Prospective Investigation of Cancer–Norfolk population-based cohort study of 13 090 participants aged 40−79. Here HCMV seropositivity was found to be associated with increased all-cause mortality, with a cause-specific analysis indicating increased HCMV seropositive HR for death from cancer (HR = 1.13, 95% CI: 0.98−1.31) [82]. While the all-cause mortality association held after adjusting for confounders, the data for cancer were not presented. Moreover, unlike all-cause mortality and death from other causes, there was not a clear relationship between HCMV antibody titre and cancer-related mortality [82], although, as per most antibody-titre analyses, interpretations can be confounded by inter-assay variability and the requirement for consistent thresholds for positivity. Notably, large differences existed according to serostatus in Townsend deprivation index, smoking status, education level and sex composition; thus, while attempts were taken to correct for these, assumptions are made of independence of confounding factors with no synergistic interaction. Perhaps most importantly, all cancers are grouped together and any granularity regarding which cancers HCMV might increase the risk of dying from was not clear. HCMV has important health implications for recipients of haematopoietic stem cell transplant (HSCT) for the treatment of haematological malignancy. HCMV reactivation following allogeneic HSCT is common and increases risk of non-relapse mortality [83]. HCMV is also a risk factor for other infections [84], with HCMV reactivation being both triggered by and, in-turn, triggering graft-versus-host disease [85]. Given these associations, HCMV is routinely assessed in the work-up for HSCT and SOTs. As such, longitudinal analyses of HSCT and SOT patients can provide insights into putative oncogenic activity of HMCV, while obviating many of the confounding factors that limit most observational HCMV epidemiology—although by necessity these studies focus on people with a degree of immunosuppression. Notably, early HCMV reactivation after HSCT is associated with reduced relapse rate for both adult and paediatric leukaemias [86–88]. It should be noted that typically the protective effect of HCMV infection from disease relapse did not outweigh increased mortality due to HCMV in this population. The anti-leukaemic effect presumably relates to HCMV-mediated immune effects, including expansion of cytotoxic T-cell clones from positive donors. In the SOT setting, a small study of tumour development post-kidney transplant demonstrated an HCMV protective activity against de novo cancer during follow-up—this protection being attributed to HCMV induction of Vδ1 γδ T cells [89]. The epidemiological findings of this small study are supported by data analysed from the United States Scientific Registry of Transplant Recipients (SRTR) across 138 112 recipients of SOT. This found no association between HCMV serostatus of donor or recipient and overall post-transplant cancer risk, in spite of immunosuppression [90]. Furthermore, this study found HCMV infection was protective against small intestine tumours and diffuse large B-cell lymphomas (DLBCLs). The protective effect of HCMV against DLBCLs was confined to those who were EBV positive. Overall, this study presents a rare example of exploration of HCMV : cancer associations across a large, well phenotyped population, which does not support HCMV promoting cancer development.

## 4. Human cytomegalovirus and cancer

### (a) Direct effects of cytomegalovirus on tumour cells

HCMV has been reported to be detected within tumours from diverse cancer types, including colorectal [91], prostate [92], breast [93], ovarian [94] and glioblastoma multiforme (GBM) [95,96]. The implications of tumoural HCMV detection for the cancer aetiology, progression and clinical outcomes are unclear, however. For example, in a study of the relationship between HCMV and ovarian cancer, 89% of patients with ovarian cancer demonstrated staining for HCMV within the tumours, whereas only 71% of patients were HCMV IgG seropositive. Moreover, while HCMV seropositivity rates were similar in patients and healthy controls, anti-HCMV IgG titres were higher in patients. Conversely, elevated patient HCMV IgG titre was associated with prolonged survival [94]. In the context of glioma and GBM, the case for a role of HCMV is potentially strongest. In addition to detection of HCMV, expression of the HCMV antigen pp65 has also been described within tumours [95], and not in uninvolved brain tissue. Most notably, these observations led to experimental work demonstrating prolonged survival in GBM patients receiving a pp65-targeted vaccine [97]. As yet, however, the clinical relevance of tumour HCMV infection to chemotherapeutic cancer treatments is not formally established. In a retrospective analysis of a cohort of patients with GBM, those receiving valganciclovir to target HCMV alongside standard-of-care treatment had prolonged survival compared with historical-control non-recipient patients [98]. However, this study’s results may be confounded by valganciclovir only being started in the absence of progressive disease on standard treatment, while immortal-time bias may influence results when comparing patients who had received a minimum period of treatment. Thus, while the study is suggestive that targeting HCMV may be of therapeutic benefit in GBM, valganciclovir has not been assessed in a placebo-controlled trial for GBM, and its efficacy in a larger patient population and suitability for standard-of-care treatment is currently unproven, although a further VIGAS2 trial has been approved, which may provide further evidence here (EudraCT 2019-001083-30). In keeping with HCMV tissue tropism, the discovery of high rates of HCMV-DNA in GBM samples might reflect the high macrophage and myeloid-derived microglial component of GBM [99]. Detection of HCMV DNA and staining in tumour samples have not always been replicated, leading to concerns over methodological consistency [100–102]. Moreover, few tumour subtypes have detectable HCMV DNA in large sequencing analyses [102,103]. While this may indicate inadequacy in the depth-of-sequencing performed to detect the low levels of HCMV infection, there is currently limited evidence to strongly support HCMV as a common, direct viral cause of cancer, although the evidence for GBM is suggestive. Recently, the International Agency for Research on Cancer (IARC) met to complete their assessment of the role of a number of viruses in the development of cancer—one of which was HCMV. While the full monograph of their assessment will be published shortly, a preliminary report of the consensus from this evaluation stated that the IARC surmised that there is ‘limited’ evidence that HCMV may cause acute lymphoblastic leukaemia. However, the IARC concluded that there was ‘inadequate’ evidence to currently support a role for HCMV in other solid tumour types. Again, this was mainly attributable to the absence of consistent observations across studies as well as to potential confounding factors [104]. Nonetheless, this does not preclude indirect relationships between HCMV infection and cancer secondary to involvement of HCMV in non-cancer cells within the tumour microenvironment (TME), and indeed such a relationship may explain many observations of HCMV within tumours.

### (b) Human cytomegalovirus and the tumour microenvironment

As discussed, the relative infidelity of HCMV in terms of tissue tropism—in particular, the propensity for HCMV to infect endothelial cells and fibroblasts, core components of the microenvironment of many solid tumours—means that generic activity of HCMV might extend across cancer subtypes. Any such generalized effects of HCMV would presumably relate to the composite cells of the TME and expression of HCMV-dependent cell-surface receptors [105]. The vascular endothelium is crucial for tumour growth and metastasis, and the enrichment of HCMV-directed cytotoxic clones in the vascular system controlling latently infected endothelial cells implies potential relevance to the process of haematogenous metastasis [32]. In keeping with this, in a murine model of breast cancer, prior MCMV inoculation was shown to alter the phenotype of seeded tumours, as well as increase the number of pulmonary metastases [106]. The ability of HCMV to infect myeloid cells additionally means that presence of HCMV in more aggressive tumours may represent intra-tumoural trafficking of HCMV-infected myeloid lineage cells, differentiating into tumour-associated macrophages (TAMs), as opposed to causal disease involvement. While inflammation may trigger HCMV reactivation, local HCMV-mediated immune activity may have pro or tumour-antagonistic activity. Consistent with the latter, several murine cancer models have demonstrated anti-cancer effects of MCMV secondary to anti-MCMV immunity. This was noted in a murine study of MCMV and a hepatotropic lymphoma, with MCMV being observed to induce apoptosis of the lymphoma cells [107]. In follow-up work, it was shown that this effect also occurred in the absence of direct exposure of tumour cells to MCMV and was related to release of the cytokine IL-15 [108], which is crucial for the survival of tissue-resident MCMV inflationary T cells [109]. A subsequent exploration of MCMV as an oncolytic virus in a murine model of melanoma unexpectedly demonstrated that intra-tumoural MCMV infection synergized with the anti-cancer effect of anti-PD-L1 therapy [110]. Intriguingly, the anti-tumour effect was related to monocyte recruitment and differentiation into M1 macrophages [111]. Thus, in animal models both pro- and anti-tumour effects of MCMV are observed. This variation likely relates to the experimental set-up—such as MCMV strain, inbred mice type and the tumour subtype examined. All these factors are rather distinct from the situation in humans, where more recent analyses of T-cell reactivity within the TME have revealed that a surprisingly small fraction of tumour-resident T cells appear to be reactive to cancer-derived antigens [5,112]. Instead, many of these T cells are ‘bystander’ clones, with undetermined antigen reactivity or TCRs known to cross-react with pathogens, in particular EBV and HCMV [5,113]. Thus, it might be envisaged that the activated adaptive immune response to HCMV infection negatively impacts tumorigenesis in humans. In keeping with this, recent evidence from our group suggests that HCMV plays an unanticipated role in the epidemiology of MM, influencing both development and onset of the disease. Specifically, we observed lower seroprevalence of HCMV in patients with MM than in control samples from the UK Biobank (OR 0.52, *p* = 0.00018 for metastatic disease, age- and sex-matched) [7]. Of particular note, this effect was larger in patients with *BRAF*-mutated melanoma, accounting for *ca* 40% of all MM, which has a more aggressive phenotype, with distinct immune interactions, propensity for haematogenous spread and the development of brain metastases [114]. Interestingly, epidemiological studies show melanoma *BRAF* mutation frequency varies markedly between geographical regions that have similar levels of UV exposure, which is striking for a malignancy largely driven by UV-mediated DNA damage, suggesting a role for other environmental factors [115]. Notably, we found patients seropositive for HCMV demonstrated delayed onset of wild-type MM (by *ca* 9 years), whereas such an effect was not noted in *BRAF*-mutated disease. Conversely, we observed fewer *BRAF-*mutated MMs in HCMV positive patients than expected, suggesting a preventive role against melanoma with this mutation [7].

### (c) Human cytomegalovirus and cancer immunotherapy

**Figure 2.**
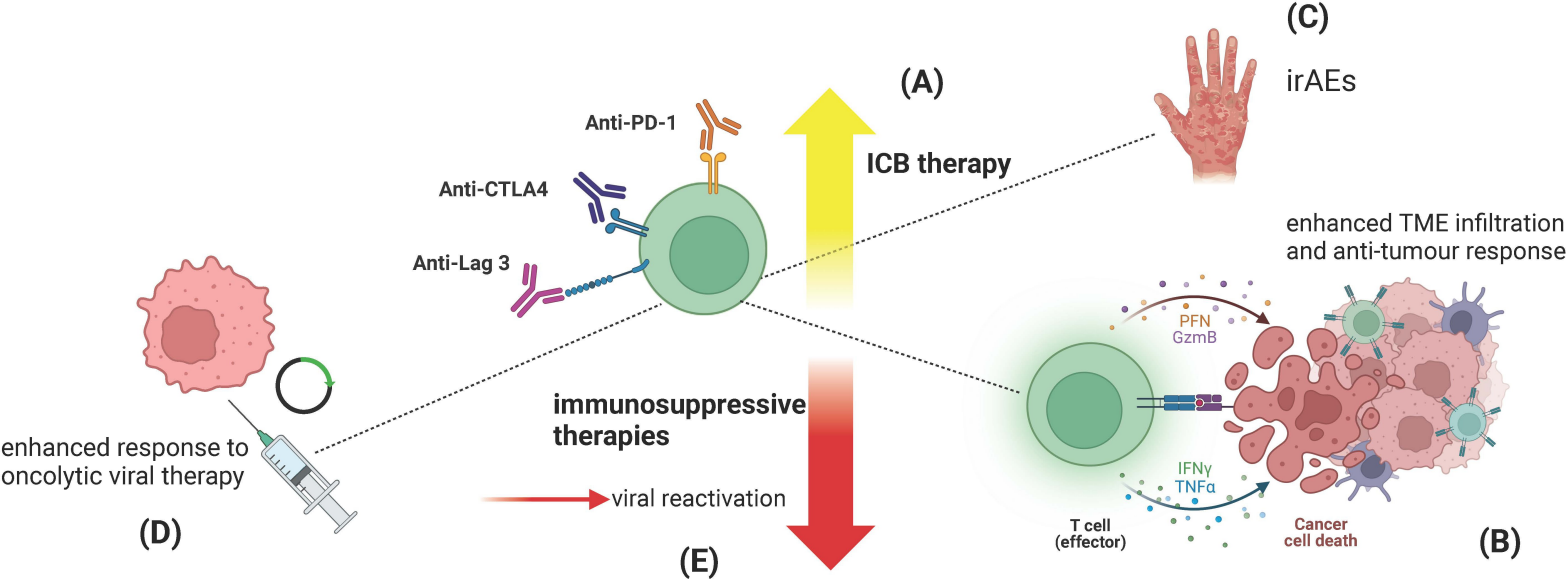
Interaction of human cytomegalovirus (HCMV) with immunotherapy response and toxicity. (A) HCMV has a positive impact on survival for patients with melanoma treated with anti-PD-1 monotherapy, while there is no clear benefit for patients receiving combination anti-CTLA-4 plus anti-PD-1 [7]. (B) Immune checkpoint blockade (ICB) therapy reinvigorates exhausted, tumour-targeting cytotoxic T cells, allowing a sustained immune destruction of tumour tissue. The presence of HCMV-reactive cytotoxic T cells appears to enhance this effect. (C) The ICB response also frequently elicits off-tumour T-cell targeting, producing autoimmune-like immune‑related adverse events (irAEs). HCMV seropositivity dramatically reduces the risk of certain irAEs such as colitis and pneumonitis, both of which carry significant morbidity and require sustained immunosuppression over weeks to control. (D) Oncolytic viral vaccine therapies often use Herpesviridae; hence HCMV presents an attractive target both as a viral vector and as a potential adjuvant for combination with ICB. (E) HCMV reactivation and associated organ-specific pathology is a known but rare complication of immunosuppressive therapy used for treating irAEs. Figure created in BioRender.

Comparatively little is known as to any relationship between HCMV infection and response to cancer immunotherapies. ICB is the central immunotherapy in clinical use and has greatly advanced the management of a wide range of cancers across both metastatic and adjuvant (i.e. preventive) settings. ICB consists of antibody-based therapies that interfere with regulatory immune cell receptors, which, upon ligation, act as inhibitory ‘checkpoints’ to control the magnitude and specificity of T-cell immune responses. The main ICB therapies in current use target the T-cell inhibitory receptor PD-1, induced on activated and exhausted T cells, or its ligand PD-L1. Other ICB treatments include ipilimumab, which blocks the protein CTLA-4—CTLA-4 being induced and trafficked to the surface of activated T cells, where it abrogates T-cell co-stimulatory signals, as well as being basally expressed on regulatory T cells (T_regs_). Finally, more recently introduced is relatlimab, which targets the inhibitory receptor LAG-3, which is expressed on ‘exhausted’ T cells. Both ipilimumab and relatlimab are given in conjunction with anti-PD-1 treatment (nivolumab), and the PD-1 : PD-L1 axis forms the core target of all ICB therapies. Treatment with ICB acts to reinvigorate anti-tumour cytotoxic T-cell activity, which is lost during the development of T-cell exhaustion [116]. Unlike chemotherapy or radiotherapy, ICB is not immunosuppressive, with ICB-induced CD8${,}^{+}$ T-cell genes being enriched for anti-viral pathways [117], highlighting the importance of a T-cell anti-viral response to ICB treatment. Given the increasing recognition that successful ICB response is shaped by non-tumour-related systemic immunity [118], the immuno-regulatory effects of HCMV may modulate ICB-induced anti-tumour immune activity. This could occur through HCMV-specific T-cell clones that demonstrate cross-reactivity with tumour antigen, or directly targeted HCMV-specific T cells by tumour-expressed HCMV antigens (figure 2). Conversely, any mechanisms of interaction may be indirect and not involve TCR recognition, but reflect divergences in T-cell responses, with enhanced cytotoxicity secondary to HCMV. Given the large populations of HCMV-specific T cells, even very minimal cross-reactivity might be anticipated to have significant effects.

One study that explored this in the context of ICB treatment of advanced lung cancer noted that patients with HCMV had increased numbers of circulating senescent CD8${,}^{+}$CD57${,}^{+}$ T cells [119]. HCMV seropositivity was associated with worse progression-free but not overall survival (OS). On further analysis, the presence of increased senescent T-cell counts appeared to drive the association—more of these cells being associated with reduced OS independent of HCMV status [119]. While the study was notable in being one of the few that addressed HCMV in the context of ICB treatment, the key finding was the association of increased senescent T-cell counts with worse OS rather than a direct association with HCMV.

Treatment of MM can consist of anti-PD-1 either alone or in conjunction with either CTLA-4- or LAG-3-targeted treatments. A potential positive role for HCMV in response to ICB was first noted in a single-cell transcriptomic study of peripheral immune responses in 40 patients with MM who had undergone treatment with combination anti-LAG-3/anti-PD-1 immunotherapy [120]. The authors found patients with higher baseline T-cell clonality and counts of adaptive NK cells responded more favourably to treatment, which induced greater upregulation of pathways associated with response to IFNγ [120]. Although survival was not explored, these phenotypes and overall response were linked with HCMV status. Thus, this intriguing work not only linked HCMV to positive ICB responses in melanoma but implicated NK cells in this process.

Combination anti-PD-1/anti-CTLA-4 treatment has strong phase III data supporting improved OS [121] and is optimal for treatment of brain metastases which are frequent in MM [122]; as such, combination anti-PD-1/anti-CTLA-4 forms standard-of-care treatment. Combination treatment, however, leads to high rates of immune-related adverse events (irAEs), with approximately 60% of patients developing severe Grade 3+ irAEs. These side-effects occur secondary to treatment-induced autoimmunity. Only 10–15% of recipients of anti-PD-1 treatment alone develop severe irAEs, with a significant proportion of these patients having similar benefit to those receiving combination treatment. For this reason, identifying patients who can be treated with anti-PD-1 alone versus those who require more intensive combination treatment is an area of active research. In recent work, we found MM patients receiving ICB had favourable pre-treatment haematological markers if they were seropositive for HCMV, with a lowered neutrophil/lymphocyte ratio (NLR), shown to be a predictive marker of ICB-treatment response, in HCMV positive patients. Furthermore, we found that HCMV positive recipients of anti-PD-1 alone had a significantly lower risk of death over follow-up than HCMV− patients (*n* = 75, HR = 0.51, 95% CI: 0.5−2.98, *p* = 0.039), with a multivariable analysis taking into account age, sex and presence of a *BRAF* mutation, finding an HR of 0.37 for death (*p* = 0.0089), while there was no association in those receiving combination treatment [7], which had similar results regardless of HCMV status. This divergence implies an adjuvant role for HCMV in recipients of anti-PD-1, essentially mimicking the effect of additional anti-CTLA-4, which was reflected in transcriptomic profiles of CD8^+^ T cells. Given the described delay in MM development in HCMV positive patients, we postulate that the cytotoxic effector memory phenotype of HCMV positive T-cell clones contributes to an enhanced endogenous cancer response prior to ICB, as well synergizing with anti-PD-1 effect during ICB treatment.

#### (i) Human cytomegalovirus infection and development of immune-related adverse event

The development of irAEs is a prominent complication of ICB therapy, causing morbidity, occasional mortality and a high burden on healthcare resources. While irAEs tend to be associated with favourable treatment outcomes [123,124], excellent oncological responses can occur in their absence, and approaches to avoid or mitigate irAEs are urgently sought. The relationship between HCMV infection and irAE development has not been explored until recently. Given TNF-mediated inflammation can elicit CMV reactivation, it is plausible that HCMV reactivation might occur directly secondary to ICB treatment. Indeed, early studies described the observation of HCMV colitis in a subset of patients with ICB related colitis that was unresponsive to standard corticosteroid treatment [125]. In general, it appears HCMV colitis is more commonly observed in patients who are already receiving corticosteroids to treat ICB colitis as opposed to being an initial presentation post-ICB treatment—thus it is often not possible to discern whether HCMV colitis was initiated directly by ICB, or secondary to immunosuppression to treat the initial presentation. In our clinical experience, HCMV reactivation is unusual in the context of initial irAE colitis, although is observed in patients failing standard management. Nonetheless, there are clear descriptions of ICB-triggered HCMV reactivation in the absence of immunosuppression—interestingly, a number of case-reports describe HCMV-associated gastritis [126–128], although ICB-induced HCMV reactivation is reported to affect other organs, including the lungs [129]. These cases are in the minority, however, with HCMV reactivation being a rare underlying cause of toxicity post-ICB, although, given diagnosis typically requires histopathological confirmation as a viraemia is unusual, it is likely many cases are missed and the true incidence is under-reported. The influence of HCMV seropositivity on developing irAEs independent of reactivation has been a focus of recent interest. One study addressing the development of irAE hepatitis in patients receiving ICB for melanoma found an association between HCMV reactivation over winter, putatively triggered by metastatic disease or intercurrent unrelated infections, and the expansion of CD4${,}^{+}$ T_EM_ cells. This T cell subset was proposed to drive hepatic injury following ICB treatment [130]. Using expansion of CD4${,}^{+}$ T_EM_ populations, in combination with HCMV status, the authors predicted hepatitis risk, although this was not linked to HCMV seropositivity alone. Moreover, the measure was not predictive of colitis or thyroiditis—indicating mechanisms diverge according to irAE type, as well as potentially cancer type and stage [130]. A recent study in melanoma patients receiving ICB was unable to replicate these findings, however, although this work did not use a clinical threshold to define HCMV seropositivity, but focused upon overall titre of HCMV IgG; the authors further finding that high anti-HCMV IgG was associated with poorer prognosis [131]. Given the non-normal distribution of HCMV IgG and the capture of HCMV negative and positive patients in the low IgG-titre group, the relevance of these findings is currently unclear. In our analysis of the relationship between ICB response in MM and HCMV status, we also explored associations between HCMV serostatus and irAE development [7]. A major bias in analyses of time-dependent variables that compete with risk of participant removal from a study is a false association due to premature study removal. In the case of irAEs, patients who succumb to disease early are not able to develop irAEs; if this is not accounted for, then toxicity may be inappropriately construed to be causally protective. To overcome this, we employed a univariate semi-competing risks model (the competing risk being death) in 308 patients treated with ICB for melanoma in either the metastatic or adjuvant setting, exploring the relationship between HCMV status and irAE development. While no association between HCMV seropositivity and less severe (Grades 1 and 2) irAEs was observed, HCMV was associated with delayed onset and lower overall incidence of severe Grade 3+ irAEs (0.52 versus 0.30 at 6 months, *p* = 2.2 × 10^−5^). This was robust to multivariable analysis controlling for age, sex, ICB type, *BRAF* status and treatment intent. Interestingly, this reduction was specific to certain organs—the largest reduction in toxicity being due to colitis mainly in recipients of combination anti-CTLA-4/anti-PD-1 treatment, with reductions also noted in myalgia and pneumonitis. Interestingly, HCMV positive patients had an increased risk of skin toxicity—although this tended to be mild, which might reflect underlying associations between skin inflammation and HCMV serostatus [132]. The immunological basis behind this is unclear, but HCMV upregulates *ZNF683* expression, which encodes a transcription factor expressed in resident memory T cells [7,133,134]; thus, it might be that HCMV-induced alterations in resident memory populations, previously associated with ICB-related colitis [135], are important. It is also likely that the reduction in TCR naivety, which has been associated with ICB irAEs [136], plays a role in reduced irAE development. Further tissue-specific studies are required to examine this immunological relationship more closely.

Overall, these findings suggest a potential protective role for HCMV, if only in certain irAE contexts. Further research into the association between HCMV infection and other T-cell subsets, especially atypical T cells, including γδ subsets, in irAE development is warranted to identify the mechanistic basis of HCMV modulation of irAE development. Some of these findings may have a translational impact in the setting of ICB use for MM. HCMV status could be envisaged to guide clinicians as to the likelihood of an individual patient developing irAEs. Furthermore, the management of irAEs, which is currently corticosteroid-based, with limited evidence supporting which patients should receive second-line immunosuppressants, could be informed by HCMV status. While more work is required, if, as well as altering the prevalence of irAEs, HCMV is found to alter their severity, then management strategies may be best tailored accordingly.

## 5. Conclusions

There is a pleiotropic interplay between HCMV status, host immunity, cancer development and the response to treatment. It remains a challenge to dissect these interactions alongside the known sociodemographic associations with HCMV. In the context of the classical immunosuppressive cancer treatments of chemotherapy and radiotherapy, HCMV is a consistent complicating factor for treatment that, if reactivated, may contribute to patient morbidity. Conversely, the evidence does not support such a deleterious effect of HCMV in the setting of ICB treatment or other pro-immune stimulatory therapies. Notably, there are increasing data to support roles for HCMV status in the oncological response to ICB as well as the development of irAEs. It is important to further elucidate the mechanistic basis of these observations; are they the indirect consequence of the large-scale changes HCMV infection enforces in the immune system, or are there direct effects that may involve HCMV reactivation and positive feedback in responses? Age differences, toxicities and other patient-specific factors such as sex and immune status play critical roles in determining cancer and ICB therapy outcomes. Not only does age appear to influence whether HCMV has beneficial or deleterious effects on immunity in the context of infection, but it is also a crucial determinant of cancer development. In addition, HCMV may have cancer-distinct effects: HCMV appears detrimental in GBM but potentially favourable in melanoma for example. These divergent qualities have clinical implications for ICB therapy and, in the case of melanoma, tailoring treatment regimens. It is also important to disentangle the virus-specific immune response that has evolved to form a targeted response tailored to that pathogen, from a virus-dependent albeit cancer-specific response, which might vary strongly depending on both affected cells and surrounding stroma. The question remains of how we can integrate patient characteristics into standard-of-care treatment and identify reliable biomarkers that reflect the immune system’s state upon treatment initiation.

In cancers where HCMV has negative associations such as GBM, systematic investigations of anti-viral therapy in a late-phase setting are obligatory to robustly test and replicate previous smaller early-stage trial data. In the context of cancer immunotherapy, achieving optimal outcomes involves a delicate balance between stimulating the immune system to maximize anti-cancer benefits, while preventing harmful autoimmunity. Given the associations of HCMV with this response in ICB treatment, further investigations into the role of HCMV-specific T cells in cancer are vital. These include employing models to functionally analyse immune cell subsets within tumours and adjacent tissues with reference to HCMV serostatus, and investigating how the modulatory effects of HCMV may be leveraged to alter clinical outcomes, ideally without HCMV infection. Given the very large populations of anti-HCMV T cells, specific stimulation of these through HCMV-specific TCRs could also be envisaged to enhance ICB-induced tumour immunity based on an endogenous immune response. Future research should further aim to deconvolute seemingly contradictory findings by stratifying according to cancer type and stage, while taking into account other host factors. It is hoped such an approach will facilitate the development of more personalized treatment strategies, tailoring for variation in systemic immunity including that due to HCMV status.

## Data Availability

This article has no additional data.

## References

[1] Hiam-Galvez KJ, Allen BM, Spitzer MH. 2021 Systemic immunity in cancer. Nat. Rev. Cancer **21**, 345–359. (10.1038/s41568-021-00347-z) 33837297 PMC8034277

[2] Mellman I, Coukos G, Dranoff G. 2011 Cancer immunotherapy comes of age. Nature **480**, 480–489. (10.1038/nature10673) 22193102 PMC3967235

[3] Klenerman P, Oxenius A. 2016 T cell responses to cytomegalovirus. Nat. Rev. Immunol. **16**, 367–377. (10.1038/nri.2016.38) 27108521

[4] Muntasell A, Vilches C, Angulo A, López‐Botet M. 2013 Adaptive reconfiguration of the human NK‐cell compartment in response to cytomegalovirus: a different perspective of the host‐pathogen interaction. Eur. J. Immunol. **43**, 1133–1141. (10.1002/eji.201243117) 23552990

[5] Simoni Y *et al*. 2018 Bystander CD8^+^ T cells are abundant and phenotypically distinct in human tumour infiltrates. Nature **557**, 575–579. (10.1038/s41586-018-0130-2) 29769722

[6] Lucibello F *et al*. 2024 Divergent local and systemic antitumor response in primary uveal melanomas. J. Exp. Med. **221**, e20232094. (10.1084/jem.20232094) 38563818 PMC10986814

[7] Milotay G *et al*. 2025 CMV serostatus is associated with improved survival and delayed toxicity onset following anti-PD-1 checkpoint blockade. Nat. Med. **31**, 2350–2364. (10.1038/s41591-025-03647-1) 40269332 PMC12283384

[8] Ballegaard V, Brændstrup P, Pedersen KK, Kirkby N, Stryhn A, Ryder LP, Gerstoft J, Nielsen SD. 2018 Cytomegalovirus-specific T-cells are associated with immune senescence, but not with systemic inflammation, in people living with HIV. Scient. Rep. **8**, 3778. (10.1038/s41598-018-21347-4) PMC5830877 29491459

[9] Gerber SI, Belval BJ, Herold BC. 1995 Differences in the role of glycoprotein C of HSV-1 and HSV-2 in viral binding may contribute to serotype differences in cell tropism. Virology **214**, 29–39. (10.1006/viro.1995.9957) 8525631

[10] Sinzger C, Digel M, Jahn G. 2008 Cytomegalovirus cell tropism. In Human cytomagalovirus (eds TE Shenk, MF Stinski), pp. 63–83, vol. 325. Berlin, Germany: Springer. (10.1007/978-3-540-77349-8_4) 18637500

[11] Nguyen C, Kamil J. 2018 Pathogen at the gates: human cytomegalovirus entry and cell tropism. Viruses **10**, 704. (10.3390/v10120704) 30544948 PMC6316194

[12] Berntsson M, Dubicanac L, Tunbäck P, Ellström A, Löwhagen G, Bergström T. 2013 Frequent detection of cytomegalovirus and Epstein–Barr virus in cervical secretions from healthy young women. Acta Obstet. Gynecol. Scand. **92**, 706–710. (10.1111/aogs.12134) 23550605

[13] Bentz GL, Jarquin-Pardo M, Chan G, Smith MS, Sinzger C, Yurochko AD. 2006 Human cytomegalovirus (HCMV) infection of endothelial cells promotes naive monocyte extravasation and transfer of productive virus to enhance hematogenous dissemination of HCMV. J. Virol. **80**, 11539–11555. (10.1128/JVI.01016-06) 16987970 PMC1642592

[14] Pachnio A, Ciaurriz M, Begum J, Lal N, Zuo J, Beggs A, Moss P. 2016 Cytomegalovirus infection leads to development of high frequencies of cytotoxic virus-specific CD4+ T cells targeted to vascular endothelium. PLOS Pathog. **12**, e1005832. (10.1371/journal.ppat.1005832) 27606804 PMC5015996

[15] Söderberg C, Larsson S, Bergstedt-Lindqvist S, Möller E. 1993 Definition of a subset of human peripheral blood mononuclear cells that are permissive to human cytomegalovirus infection. J. Virol. **67**, 3166–3175. (10.1128/jvi.67.6.3166-3175.1993) 7684461 PMC237655

[16] Schwartz M, Stern‐Ginossar N. 2023 Rethinking human cytomegalovirus latency reservoir. Ann. N. Y. Acad. Sci. **1524**, 30–36. (10.1111/nyas.14994) 37026581

[17] Jackson J, Sparer T. 2018 There is always another way! cytomegalovirus’ multifaceted dissemination schemes. Viruses **10**, 383. (10.3390/v10070383) 30037007 PMC6071125

[18] Cobbs C. 2019 Cytomegalovirus is a tumor-associated virus: armed and dangerous. Curr. Opin. Virol. **39**, 49–59. (10.1016/j.coviro.2019.08.003) 31525538

[19] Lachmann R *et al*. 2018 Cytomegalovirus (CMV) seroprevalence in the adult population of Germany. PLoS One **13**, e0200267. (10.1371/journal.pone.0200267) 30044826 PMC6059406

[20] Lopo S, Vinagre E, Palminha P, Paixao MT, Nogueira P, Freitas MG. 2011 Seroprevalence to cytomegalovirus in the Portuguese population, 2002-2003. Euro Surveill. **16**, 19896. (10.2807/ese.16.25.19896-en) 21722611

[21] Olsson J, Kok E, Adolfsson R, Lövheim H, Elgh F. 2017 Herpes virus seroepidemiology in the adult Swedish population. Immun. Ageing **14**, 6. (10.1186/s12979-017-0093-4) 28491117 PMC5424393

[22] Ng SH, Puong KY, Ng W, Wan WY. 2024 Seroprevalence of cytomegalovirus over the last 2 decades (2001–2020): a retrospective data analysis from a single laboratory in Singapore. Ann. Acad. Med. Singap. **53**, 396–398. (10.47102/annals-acadmedsg.2023363) 38979996

[23] Shigemi D, Yamaguchi S, Otsuka T, Kamoi S, Takeshita T. 2015 Seroprevalence of cytomegalovirus IgG antibodies among pregnant women in Japan from 2009-2014. Am. J. Infect. Control **43**, 1218–1221. (10.1016/j.ajic.2015.06.026) 26277571

[24] Mussi-Pinhata MM, Yamamoto AY. 2020 Natural history of congenital cytomegalovirus infection in highly seropositive populations. J. Infect. Dis. **221**, S15–S22. (10.1093/infdis/jiz443) 32134482 PMC7057789

[25] Sunil-Chandra NP, Jayasundara MVML, Gunathilaka MGRSS, Suminda SDH. 2025 Human cytomegalovirus (HCMV) trends in Sri Lanka: insights from a hospital-based seroprevalence analysis. BMC Infect. Dis. **25**, 184. (10.1186/s12879-025-10594-2) 39920600 PMC11806834

[26] Fowler K *et al*. 2022 A systematic literature review of the global seroprevalence of cytomegalovirus: possible implications for treatment, screening, and vaccine development. BMC Public Health **22**, 1659. (10.1186/s12889-022-13971-7) 36050659 PMC9435408

[27] Hoehl S, Berger A, Ciesek S, Rabenau HF. 2020 Thirty years of CMV seroprevalence—a longitudinal analysis in a German university hospital. Eur. J. Clin. Microbiol. Infect. Dis. **39**, 1095–1102. (10.1007/s10096-020-03814-x) 31989374 PMC7225192

[28] Vyse A, Hesketh L, Pebody R. 2009 The burden of infection with cytomegalovirus in England and Wales: how many women are infected in pregnancy? Epidemiol. Infect. **137**, 526–533. (10.1017/S0950268808001258) 18789177

[29] Mentzer AJ *et al*. 2022 Identification of host–pathogen-disease relationships using a scalable multiplex serology platform in UK Biobank. Nat. Commun. **13**, 1818. (10.1038/s41467-022-29307-3) 35383168 PMC8983701

[30] Dunn W, Chou C, Li H, Hai R, Patterson D, Stolc V, Zhu H, Liu F. 2003 Functional profiling of a human cytomegalovirus genome. Proc. Natl Acad. Sci. USA **100**, 14223–14228. (10.1073/pnas.2334032100) 14623981 PMC283573

[31] Loewendorf A, Benedict CA. 2010 Modulation of host innate and adaptive immune defenses by cytomegalovirus: timing is everything. J. Intern. Med. **267**, 483–501. (10.1111/j.1365-2796.2010.02220.x) 20433576 PMC2902254

[32] Moss P. 2019 ‘From immunosenescence to immune modulation’: a re-appraisal of the role of cytomegalovirus as major regulator of human immune function. Med. Microbiol. Immunol. **208**, 271–280. (10.1007/s00430-019-00612-x) 31053999

[33] Compton T, Kurt-Jones EA, Boehme KW, Belko J, Latz E, Golenbock DT, Finberg RW. 2003 Human cytomegalovirus activates inflammatory cytokine responses via CD14 and toll-like receptor 2. J. Virol. **77**, 4588–4596. (10.1128/jvi.77.8.4588-4596.2003) 12663765 PMC152130

[34] Prösch S, Staak K, Stein J, Liebenthal C, Stamminger T, Volk HD, Krüger DH. 1995 Stimulation of the human cytomegalovirus IE enhancer/promoter in HL-60 cells by TNFα is mediated via induction of NF-κB. Virology **208**, 197–206. (10.1006/viro.1995.1143) 11831701

[35] Varani S, Frascaroli G, Landini MP, Söderberg‐Nauclér C. 2009 Human cytomegalovirus targets different subsets of antigen‐presenting cells with pathological consequences for host immunity: implications for immunosuppression, chronic inflammation and autoimmunity. Rev. Med. Virol. **19**, 131–145. (10.1002/rmv.609) 19367559

[36] Gkrania-Klotsas E, Langenberg C, Sharp SJ, Luben R, Khaw KT, Wareham NJ. 2012 Higher immunoglobulin G antibody levels against cytomegalovirus are associated with incident ischemic heart disease in the population-based EPIC-Norfolk Cohort. J. Infect. Dis. **206**, 1897–1903. (10.1093/infdis/jis620) 23045624

[37] Gugliesi F, Pasquero S, Griffante G, Scutera S, Albano C, Pacheco SFC, Riva G, Dell’Oste V, Biolatti M. 2021 Human cytomegalovirus and autoimmune diseases: where are we? Viruses **13**, 260. (10.3390/v13020260) 33567734 PMC7914970

[38] Varani S, Cederarv M, Feld S, Tammik C, Frascaroli G, Landini MP, Söderberg-Nauclér C. 2007 Human cytomegalovirus differentially controls B cell and T cell responses through effects on plasmacytoid dendritic cells. J. Immunol. **179**, 7767–7776. (10.4049/jimmunol.179.11.7767) 18025223

[39] Hamilton EM, Allen NE, Mentzer AJ, Littlejohns TJ. 2022 Human cytomegalovirus and risk of incident cardiovascular disease in United Kingdom biobank. J. Infect. Dis. **225**, 1179–1188. (10.1093/infdis/jiab364) 34279656 PMC8974830

[40] Yates TA, Griffith GJ, Morris TT. 2022 Human cytomegalovirus and risk of incident cardiovascular disease in UK biobank. J. Infect. Dis. **225**, 1301–1302. (10.1093/infdis/jiab571) 35024849 PMC8974855

[41] Belga S, MacDonald C, Chiang D, Kabbani D, Shojai S, Abraldes JG, Cervera C. 2021 Donor graft cytomegalovirus serostatus and the risk of arterial and venous thrombotic events in seronegative recipients after non-thoracic solid organ transplantation. Clin. Infect. Dis. **72**, 845–852. (10.1093/cid/ciaa125) 32025704

[42] Courivaud C, Bamoulid J, Chalopin JM, Gaiffe E, Tiberghien P, Saas P, Ducloux D. 2013 Cytomegalovirus exposure and cardiovascular disease in kidney transplant recipients. J. Infect. Dis. **207**, 1569–1575. (10.1093/infdis/jit064) 23417659

[43] Shimamura M. 2013 The contribution of cytomegalovirus to atherosclerotic events after kidney transplantation. J. Infect. Dis. **207**, 1487–1490. (10.1093/infdis/jit065) 23417660

[44] Hewitt EW, Gupta SS, Lehner PJ. 2001 The human cytomegalovirus gene product US6 inhibits ATP binding by TAP. EMBO J. **20**, 387–396. (10.1093/emboj/20.3.387) 11157746 PMC133477

[45] Park B, Kim Y, Shin J, Lee S, Cho K, Früh K, Lee S, Ahn K. 2004 Human Cytomegalovirus inhibits tapasin-dependent peptide loading and optimization of the MHC class I peptide cargo for immune evasion. Immunity **20**, 71–85. (10.1016/s1074-7613(03)00355-8) 14738766

[46] Ziegler H, Muranyi W, Burgert HG, Kremmer E, Koszinowski UH. 2000 The luminal part of the murine cytomegalovirus glycoprotein gp40 catalyzes the retention of MHC class I molecules. EMBO J. **19**, 870–881. (10.1093/emboj/19.5.870) 10698929 PMC305627

[47] Liu Z, Winkler M, Biegalke B. 2009 Human cytomegalovirus: host immune modulation by the viral US3 gene. Int. J. Biochem. Cell Biol. **41**, 503–506. (10.1016/j.biocel.2008.10.012) 18992841

[48] Shukla SA *et al*. 2015 Comprehensive analysis of cancer-associated somatic mutations in class I HLA genes. Nat. Biotechnol. **33**, 1152–1158. (10.1038/nbt.3344) 26372948 PMC4747795

[49] Wang D, Bresnahan W, Shenk T. 2004 Human cytomegalovirus encodes a highly specific RANTES decoy receptor. Proc. Natl Acad. Sci. USA **101**, 16642–16647. (10.1073/pnas.0407233101) 15536129 PMC534536

[50] Kotenko SV, Saccani S, Izotova LS, Mirochnitchenko OV, Pestka S. 2000 Human cytomegalovirus harbors its own unique IL-10 homolog (cmvIL-10). Proc. Natl Acad. Sci. USA **97**, 1695–1700. (10.1073/pnas.97.4.1695) 10677520 PMC26498

[51] Jenkins C, Garcia W, Godwin MJ, Spencer JV, Stern JL, Abendroth A, Slobedman B. 2008 Immunomodulatory properties of a viral homolog of human interleukin-10 expressed by human cytomegalovirus during the latent phase of infection. J. Virol. **82**, 3736–3750. (10.1128/JVI.02173-07) 18216121 PMC2268454

[52] Mumm JB *et al*. 2011 IL-10 elicits IFNγ-dependent tumor immune surveillance. Cancer Cell **20**, 781–796. (10.1016/j.ccr.2011.11.003) 22172723

[53] Guo Y *et al*. 2021 Metabolic reprogramming of terminally exhausted CD8^+^ T cells by IL-10 enhances anti-tumor immunity. Nat. Immunol. **22**, 746–756. (10.1038/s41590-021-00940-2) 34031618 PMC7610876

[54] Mason GM, Poole E, Sissons JGP, Wills MR, Sinclair JH. 2012 Human cytomegalovirus latency alters the cellular secretome, inducing cluster of differentiation (CD)4^+^ T-cell migration and suppression of effector function. Proc. Natl Acad. Sci. USA **109**, 14538–14543. (10.1073/pnas.1204836109) 22826250 PMC3437838

[55] Mason GM, Jackson S, Okecha G, Poole E, Sissons JGP, Sinclair J, Wills MR. 2013 Human cytomegalovirus latency-associated proteins elicit immune-suppressive IL-10 producing CD4^+^ T cells. PLoS Pathog. **9**, e1003635. (10.1371/journal.ppat.1003635) 24130479 PMC3795018

[56] Crawford A, Angelosanto JM, Nadwodny KL, Blackburn SD, Wherry EJ. 2011 A role for the chemokine RANTES in regulating CD8 T cell responses during chronic viral infection. PLoS Pathog. **7**, e1002098. (10.1371/journal.ppat.1002098) 21814510 PMC3141034

[57] Aldinucci D, Colombatti A. 2014 The inflammatory chemokine CCL5 and cancer progression. Mediat. Inflamm. **2014**, 292376. (10.1155/2014/292376) PMC3910068 24523569

[58] Skaletskaya A, Bartle LM, Chittenden T, McCormick AL, Mocarski ES, Goldmacher VS. 2001 A cytomegalovirus-encoded inhibitor of apoptosis that suppresses caspase-8 activation. Proc. Natl Acad. Sci. USA **98**, 7829–7834. (10.1073/pnas.141108798) 11427719 PMC35427

[59] Kim HS *et al*. 2003 Inactivating mutations of caspase-8 gene in colorectal carcinomas. Gastroenterology **125**, 708–715. (10.1016/s0016-5085(03)01059-x) 12949717

[60] Soung YH *et al*. 2005 CASPASE-8 gene is inactivated by somatic mutations in gastric carcinomas. Cancer Res. **65**, 815–821. (10.1158/0008-5472.815.65.3) 15705878

[61] Li C, Egloff AM, Sen M, Grandis JR, Johnson DE. 2014 Caspase‐8 mutations in head and neck cancer confer resistance to death receptor‐mediated apoptosis and enhance migration, invasion, and tumor growth. Mol. Oncol. **8**, 1220–1230. (10.1016/j.molonc.2014.03.018) 24816188 PMC4198498

[62] Lu HJ, Su CW, Su SC, Chang LC, Wu MF, Lin CW, Yang SF. 2025 Prognostic impact of caspase-8 mutation in oral cavity squamous cell carcinoma. Oral Dis. **31**, 769–781. (10.1111/odi.15124) 39289898

[63] Sylwester AW *et al*. 2005 Broadly targeted human cytomegalovirus-specific CD4^+^ and CD8^+^ T cells dominate the memory compartments of exposed subjects. J. Exp. Med. **202**, 673–685. (10.1084/jem.20050882) 16147978 PMC2212883

[64] van den Berg SPH, Pardieck IN, Lanfermeijer J, Sauce D, Klenerman P, van Baarle D, Arens R. 2019 The hallmarks of CMV-specific CD8 T-cell differentiation. Med. Microbiol. Immunol. **208**, 365–373. (10.1007/s00430-019-00608-7) 30989333 PMC6647465

[65] Sierro S, Rothkopf R, Klenerman P. 2005 Evolution of diverse antiviral CD8^+^ T cell populations after murine cytomegalovirus infection. Eur. J. Immunol. **35**, 1113–1123. (10.1002/eji.200425534) 15756645

[66] Snyder CM, Cho KS, Bonnett EL, Allan JE, Hill AB. 2011 Sustained CD8+ T cell memory inflation after infection with a single-cycle cytomegalovirus. PLoS Pathog. **7**, e1002295. (10.1371/journal.ppat.1002295) 21998590 PMC3188546

[67] Schober K, Buchholz VR, Busch DH. 2018 TCR repertoire evolution during maintenance of CMV‐specific T‐cell populations. Immunol. Rev. **283**, 113–128. (10.1111/imr.12654) 29664573

[68] Moss P, Khan N. 2004 CD8^+^ T-cell immunity to cytomegalovirus. Hum. Immunol. **65**, 456–464. (10.1016/j.humimm.2004.02.014) 15172445

[69] Redeker A, Welten SPM, Arens R. 2014 Viral inoculum dose impacts memory T‐cell inflation. Eur. J. Immunol. **44**, 1046–1057. (10.1002/eji.201343946) 24356925

[70] Kahan SM, Wherry EJ, Zajac AJ. 2015 T cell exhaustion during persistent viral infections. Virology **479**, 180–193. (10.1016/j.virol.2014.12.033) 25620767 PMC4424083

[71] Seckert CK *et al*. 2012 Viral latency drives ‘memory inflation’: a unifying hypothesis linking two hallmarks of cytomegalovirus infection. Med. Microbiol. Immunol. **201**, 551–566. (10.1007/s00430-012-0273-y) 22991040

[72] Samson LD *et al*. 2020 Limited effect of duration of CMV infection on adaptive immunity and frailty: insights from a 27‐year‐long longitudinal study. Clin. Transl. Immunol. **9**, e1193. (10.1002/cti2.1193) PMC7586993 33133599

[73] Pitard V, Roumanes D, Lafarge X, Couzi L, Garrigue I, Lafon ME, Merville P, Moreau JF, Déchanet-Merville J. 2008 Long-term expansion of effector/memory Vδ2^−^ γδ T cells is a specific blood signature of CMV infection. Blood **112**, 1317–1324. (10.1182/blood-2008-01-136713) 18539896 PMC2515135

[74] López-Botet M, De Maria A, Muntasell A, Della Chiesa M, Vilches C. 2023 Adaptive NK cell response to human cytomegalovirus: facts and open issues. Semin. Immunol. **65**, 101706. (10.1016/j.smim.2022.101706) 36542944

[75] Lopez-Sejas N, Campos C, Hassouneh F, Sanchez-Correa B, Tarazona R, Pera A, Solana R. 2016 Effect of CMV and aging on the differential expression of CD300a, CD161, T-bet, and eomes on NK cell subsets. Front. Immunol. **7**, 476. (10.3389/fimmu.2016.00476) 27872625 PMC5097920

[76] Kuijpers TW, Baars PA, Dantin C, van den Burg M, van Lier RAW, Roosnek E. 2008 Human NK cells can control CMV infection in the absence of T cells. Blood **112**, 914–915. (10.1182/blood-2008-05-157354) 18650467

[77] Franceschi C, Garagnani P, Parini P, Giuliani C, Santoro A. 2018 Inflammaging: a new immune–metabolic viewpoint for age-related diseases. Nat. Rev. Endocrinol. **14**, 576–590. (10.1038/s41574-018-0059-4) 30046148

[78] Pawelec G, Derhovanessian E, Larbi A. 2010 Immunosenescence and cancer. Crit. Rev. Oncol. Hematol. **75**, 165–172. (10.1016/j.critrevonc.2010.06.012) 20656212

[79] Furman D *et al*. 2015 Cytomegalovirus infection enhances the immune response to influenza. Sci. Transl. Med. **7**, 281ra43. (10.1126/scitranslmed.aaa2293) PMC4505610 25834109

[80] Quinn M, Erkes DA, Snyder CM. 2016 Cytomegalovirus and immunotherapy: opportunistic pathogen, novel target for cancer and a promising vaccine vector. Immunotherapy **8**, 211–221. (10.2217/imt.15.110) 26786895 PMC5619022

[81] Herbein G, Nehme Z. 2020 Tumor control by cytomegalovirus: a door open for oncolytic virotherapy? Mol. Ther. Oncolyt. **17**, 1–8. (10.1016/j.omto.2020.03.004) PMC7150429 32300639

[82] Gkrania-Klotsas E, Langenberg C, Sharp SJ, Luben R, Khaw KT, Wareham NJ. 2013 Seropositivity and higher immunoglobulin G antibody levels against cytomegalovirus are associated with mortality in the population-based European Prospective Investigation of Cancer–Norfolk Cohort. Clin. Infect. Dis. **56**, 1421–1427. (10.1093/cid/cit083) 23442763 PMC3634310

[83] Teira P *et al*. 2016 Early cytomegalovirus reactivation remains associated with increased transplant-related mortality in the current era: a CIBMTR analysis. Blood **127**, 2427–2438. (10.1182/blood-2015-11-679639) 26884374 PMC4874224

[84] Nichols WG, Corey L, Gooley T, Davis C, Boeckh M. 2002 High risk of death due to bacterial and fungal infection among cytomegalovirus (CMV)—seronegative recipients of stem cell transplants from seropositive donors: evidence for indirect effects of primary CMV infection. J. Infect. Dis. **185**, 273–282. (10.1086/338624) 11807708

[85] Cantoni N *et al*. 2010 Evidence for a bidirectional relationship between cytomegalovirus replication and acute graft-versus-host disease. Biol. Blood Marrow Transplant. **16**, 1309–1314. (10.1016/j.bbmt.2010.03.020) 20353832

[B86] elmaagacli ah *et al* *et al* *et al et al*. 2011 Early human cytomegalovirus replication after transplantation is associated with a decreased relapse risk: evidence for a putative virus-versus-leukemia effect in acute myeloid leukemia patients. blood **118**, 1402–1412. (10.1182/blood-2010-08-304121) 21540462

[87] Green ML, Leisenring WM, Xie H, Walter RB, Mielcarek M, Sandmaier BM, Riddell SR, Boeckh M. 2013 CMV reactivation after allogeneic HCT and relapse risk: evidence for early protection in acute myeloid leukemia. Blood **122**, 1316–1324. (10.1182/blood-2013-02-487074) 23744585 PMC3744995

[88] Inagaki J, Noguchi M, Kurauchi K, Tanioka S, Fukano R, Okamura J. 2016 Effect of cytomegalovirus reactivation on relapse after allogeneic hematopoietic stem cell transplantation in pediatric acute leukemia. Biol. Blood Marrow Transplant. **22**, 300–306. (10.1016/j.bbmt.2015.09.006) 26371373

[89] Couzi L *et al*. 2010 Cytomegalovirus-induced γδ T cells associate with reduced cancer risk after kidney transplantation. J. Am. Soc. Nephrol. **21**, 181–188. (10.1681/asn.2008101072) 19713314 PMC2799280

[90] Geris JM, Spector LG, Pfeiffer RM, Limaye AP, Yu KJ, Engels EA. 2022 Cancer risk associated with cytomegalovirus infection among solid organ transplant recipients in the United States. Cancer **128**, 3985–3994. (10.1002/cncr.34462) 36126024 PMC9633408

[B91] harkins l, volk al, samanta m, mikolaenko i, britt wj, bland ki, cobbs cs, , , , , , , , , , , , . 2002 Specific localisation of human cytomegalovirus nucleic acids and proteins in human colorectal cancer. lancet **360**, 1557–1563. (10.1016/s0140-6736(02)11524-8) 12443594

[92] Samanta M, Harkins L, Klemm K, Britt WJ, Cobbs CS. 2003 High prevalence of human cytomegalovirus in prostatic intraepithelial neoplasia and prostatic carcinoma. J. Urol. **170**, 998–1002. (10.1097/01.ju.0000080263.46164.97) 12913758

[93] Harkins LE, Matlaf LA, Soroceanu L, Klemm K, Britt WJ, Wang W, Bland KI, Cobbs CS. 2010 Detection of human cytomegalovirus in normal and neoplastic breast epithelium. Herpesviridae **1**, 8. (10.1186/2042-4280-1-8) 21429243 PMC3063230

[94] Rådestad AF, Estekizadeh A, Cui HL, Kostopoulou ON, Davoudi B, Hirschberg AL, Carlson J, Rahbar A, Söderberg-Naucler C. 2018 Impact of human cytomegalovirus infection and its immune response on survival of patients with ovarian cancer. Transl. Oncol. **11**, 1292–1300. (10.1016/j.tranon.2018.08.003) 30172882 PMC6121833

[95] Cobbs CS, Harkins L, Samanta M, Gillespie GY, Bharara S, King PH, Nabors LB, Cobbs CG, Britt WJ. 2002 Human cytomegalovirus infection and expression in human malignant glioma. Cancer Res. **62**, 3347–3350. 12067971

[96] Rahbar A, Orrego A, Peredo I, Dzabic M, Wolmer-Solberg N, Strååt K, Stragliotto G, Söderberg-Nauclér C. 2013 Human cytomegalovirus infection levels in glioblastoma multiforme are of prognostic value for survival. J. Clin. Virol. **57**, 36–42. (10.1016/j.jcv.2012.12.018) 23391370

[97] Batich KA *et al*. 2017 Long-term survival in glioblastoma with cytomegalovirus pp65-targeted vaccination. Clin. Cancer Res. **23**, 1898–1909. (10.1158/1078-0432.ccr-16-2057) 28411277 PMC5559300

[98] Söderberg-Nauclér C, Rahbar A, Stragliotto G. 2013 Survival in patients with glioblastoma receiving valganciclovir. N. Engl. J. Med. **369**, 985–986. (10.1056/nejmc1302145) 24004141

[99] Geribaldi-Doldán N, Fernández-Ponce C, Quiroz RN, Sánchez-Gomar I, Escorcia LG, Velásquez EP, Quiroz EN. 2020 The role of microglia in glioblastoma. Front. Oncol. **10**, 603495. (10.3389/fonc.2020.603495) 33585220 PMC7879977

[100] Yamashita Y *et al*. 2014 Lack of presence of the human cytomegalovirus in human glioblastoma. Mod. Pathol. **27**, 922–929. (10.1038/modpathol.2013.219) 24336154

[101] Garcia-Martinez A, Alenda C, Irles E, Ochoa E, Quintanar T, Rodriguez-Lescure A, Soto JL, Barbera VM. 2017 Lack of cytomegalovirus detection in human glioma. Virol. J **14**, 216. (10.1186/s12985-017-0885-3) 29116009 PMC5678593

[102] Zapatka M *et al*. 2020 The landscape of viral associations in human cancers. Nat. Genet. **52**, 320–330. (10.1038/s41588-019-0558-9) 32025001 PMC8076016

[103] Ge Y, Lu J, Puiu D, Revsine M, Salzberg SL. 2025 Comprehensive analysis of microbial content in whole-genome sequencing samples from the Cancer Genome Atlas project. Sci. Transl. Med. **17**, eads6335. (10.1101/2024.05.24.595788) 40901923 PMC12821378

[104] Karagas MR *et al*. 2025 Carcinogenicity of hepatitis D virus, human cytomegalovirus, and Merkel cell polyomavirus. Lancet Oncol. **26**, 994–995. (10.1016/S1470-2045(25)00403-6) PMC12281636 40587985

[105] Murray MJ, Peters NE, Reeves MB. 2018 Navigating the host cell response during entry into sites of latent cytomegalovirus infection. Pathogens **7**, 30. (10.3390/pathogens7010030) 29547547 PMC5874756

[106] Yang Z *et al*. 2019 Latent cytomegalovirus infection in female mice increases breast cancer metastasis. Cancers **11**, 447. (10.3390/cancers11040447) 30934926 PMC6520675

[107] Erlach KC, Böhm V, Seckert CK, Reddehase MJ, Podlech J. 2006 Lymphoma cell apoptosis in the liver induced by distant murine cytomegalovirus infection. J. Virol. **80**, 4801–4819. (10.1128/JVI.80.10.4801-4819.2006) 16641273 PMC1472044

[108] Erlach KC, Reddehase MJ, Podlech J. 2015 Mechanism of tumor remission by cytomegalovirus in a murine lymphoma model: evidence for involvement of virally induced cellular interleukin-15. Med. Microbiol. Immunol. **204**, 355–366. (10.1007/s00430-015-0408-z) 25805565

[109] Baumann NS *et al*. 2018 Tissue maintenance of CMV-specific inflationary memory T cells by IL-15. PLoS Pathog. **14**, e1006993. (10.1371/journal.ppat.1006993) 29652930 PMC5919076

[110] Erkes DA, Xu G, Daskalakis C, Zurbach KA, Wilski NA, Moghbeli T, Hill AB, Snyder CM. 2016 Intratumoral infection with murine cytomegalovirus synergizes with PD-L1 blockade to clear melanoma lesions and induce long-term immunity. Mol. Ther. **24**, 1444–1455. (10.1038/mt.2016.121) 27434584 PMC5023369

[111] Wilski NA, Del Casale C, Purwin TJ, Aplin AE, Snyder CM. 2019 Murine cytomegalovirus infection of melanoma lesions delays tumor growth by recruiting and repolarizing monocytic phagocytes in the tumor. J. Virol. **93**, 1128. (10.1128/jvi.00533-19) PMC6798091 31375579

[112] Scheper W *et al*. 2019 Low and variable tumor reactivity of the intratumoral TCR repertoire in human cancers. Nat. Med. **25**, 89–94. (10.1038/s41591-018-0266-5) 30510250

[113] Meier SL, Satpathy AT, Wells DK. 2022 Bystander T cells in cancer immunology and therapy. Nat. Cancer **3**, 143–155. (10.1038/s43018-022-00335-8) 35228747

[114] Castellani G, Buccarelli M, Arasi MB, Rossi S, Pisanu ME, Bellenghi M, Lintas C, Tabolacci C. 2023 BRAF mutations in melanoma: biological aspects, therapeutic implications, and circulating biomarkers. Cancers **15**, 4026. (10.3390/cancers15164026) 37627054 PMC10452867

[115] Saroufim M *et al*. 2014 BRAF analysis on a spectrum of melanocytic neoplasms: an epidemiological study across differing UV regions. Am. J. Dermatopathol. **36**, 68–73. (10.1097/DAD.0b013e318293f355) 23782679

[116] Ribas A, Wolchok JD. 2018 Cancer immunotherapy using checkpoint blockade. Science **359**, 1350–1355. (10.1126/science.aar4060) 29567705 PMC7391259

[117] Fairfax BP *et al*. 2020 Peripheral CD8^+^ T cell characteristics associated with durable responses to immune checkpoint blockade in patients with metastatic melanoma. Nat. Med. **26**, 193–199. (10.1038/s41591-019-0734-6) 32042196 PMC7611047

[118] Spitzer MH *et al*. 2017 Systemic immunity is required for effective cancer immunotherapy. Cell **168**, 487–502.(10.1016/j.cell.2016.12.022) 28111070 PMC5312823

[119] Naigeon M. 2023 Human virome profiling identified CMV as the major viral driver of a high accumulation of senescent CD8^+^ T cells in patients with advanced NSCLC. Sci. Adv. **9**, eadh0708. (10.1126/sciadv.adh0708) 37939189 PMC10631735

[120] Huuhtanen J *et al*. 2023 Single-cell characterization of anti-LAG-3 and anti-PD-1 combination treatment in patients with melanoma. J. Clin. Invest. **133**, e164809. (10.1172/JCI164809) 36719749 PMC10014104

[121] Wolchok JD *et al*. 2025 Final, 10-year outcomes with nivolumab plus ipilimumab in advanced melanoma. N. Engl. J. Med. **392**, 11–22. (10.1056/nejmoa1302369) 39282897 PMC12080919

[122] Tawbi HA *et al*. 2021 Long-term outcomes of patients with active melanoma brain metastases treated with combination nivolumab plus ipilimumab (CheckMate 204): final results of an open-label, multicentre, phase 2 study. Lancet Oncol. **22**, 1692–1704. (10.1016/s1470-2045(21)00545-3) 34774225 PMC9328029

[123] Zhou X, Yao Z, Yang H, Liang N, Zhang X, Zhang F. 2020 Are immune-related adverse events associated with the efficacy of immune checkpoint inhibitors in patients with cancer? A systematic review and meta-analysis. BMC Med. **18**, 87. (10.1186/s12916-020-01549-2) 32306958 PMC7169020

[124] Ye W *et al*. 2021 Checkpoint-blocker-induced autoimmunity is associated with favourable outcome in metastatic melanoma and distinct T-cell expression profiles. Br. J. Cancer **124**, 1661–1669. (10.1038/s41416-021-01310-3) 33723392 PMC8110747

[125] Franklin C *et al*. 2017 Cytomegalovirus reactivation in patients with refractory checkpoint inhibitor-induced colitis. Eur. J. Cancer **86**, 248–256. (10.1016/j.ejca.2017.09.019) 29055840

[126] Lu J, Firpi-Morell RJ, Dang LH, Lai J, Liu X. 2018 An unusual case of gastritis in one patient receiving PD-1 blocking therapy: coexisting immune-related gastritis and cytomegaloviral infection. Gastroenterol. Res. **11**, 383–387. (10.14740/gr1068w) PMC6188031 30344812

[127] Indini A, Gueli R, Cerati M, Rijavec E, Parravicini M, Casagrande S, Rovelli C, Grossi PA, Grossi F. 2024 Cytomegalovirus gastritis as a rare adverse event during combined ipilimumab and nivolumab in a patient with melanoma. Melanoma Res. **34**, 386–389. (10.1097/CMR.0000000000000981) 38768445

[128] Anastasopoulou A, Samarkos M, Diamantopoulos P, Vourlakou C, Ziogas DC, Avramopoulos P, Kouzis P, Haanen J, Gogas H. 2023 Cytomegalovirus infections in patients treated with immune checkpoint inhibitors for solid malignancies. Open Forum Infect. Dis. **10**, ofad164. (10.1093/ofid/ofad164) 37065986 PMC10099470

[129] Badran O, Ouryvaev A, Baturov V, Shai A. 2021 Cytomegalovirus pneumonia complicating immune checkpoint inhibitors‑induced pneumonitis: a case report. Mol. Clin. Oncol. **14**, 120. (10.3892/mco.2021.2282) 33903826 PMC8060847

[130] Hutchinson JA *et al*. 2021 Virus-specific memory T cell responses unmasked by immune checkpoint blockade cause hepatitis. Nat. Commun. **12**, 1439. (10.1038/s41467-021-21572-y) 33664251 PMC7933278

[131] Strobel SB, Machiraju D, Wiecken M, Richter J, Klein JAF, Berger A, Hassel JC. 2025 CMV IgG in the blood is not associated with hepatitis but correlates with poor outcomes in immunotherapy treated melanoma patients. Cancer Immunol. Immunother. **74**, 59. (10.1007/s00262-024-03859-3) 39751902 PMC11699187

[132] Lape M *et al*. 2024 After the infection: a survey of pathogens and non-communicable human disease. medRxiv, 2023-09. (10.1101/2023.09.14.23295428)

[133] Hertoghs KML *et al*. 2010 Molecular profiling of cytomegalovirus-induced human CD8+ T cell differentiation. J. Clin. Investig. **120**, 4077–4090. (10.1172/jci42758) 20921622 PMC2964975

[134] Parry EM *et al*. 2023 ZNF683 marks a CD8^+^ T cell population associated with anti-tumor immunity following anti-PD-1 therapy for Richter syndrome. Cancer Cell **41**, 1803–1816.(10.1016/j.ccell.2023.08.013) 37738974 PMC10618915

[135] Sasson SC *et al*. 2021 Interferon-gamma–producing CD8^+^ tissue resident memory T cells are a targetable hallmark of immune checkpoint inhibitor–colitis. Gastroenterology **161**, 1229–1244.(10.1053/j.gastro.2021.06.025) 34147519 PMC8527886

[136] Lozano AX *et al*. 2022 T cell characteristics associated with toxicity to immune checkpoint blockade in patients with melanoma. Nat. Med. **28**, 353–362. (10.1038/s41591-021-01623-z) 35027754 PMC8866214

